# Dentists’ knowledge, attitudes and practices regarding Hepatitis B and C and HIV/AIDS in Sanandaj, Iran

**DOI:** 10.1186/s12903-018-0685-1

**Published:** 2018-12-18

**Authors:** Masomeh Rostamzadeh, Abdorrahim Afkhamzadeh, Sirus Afrooz, Kaveh Mohamadi, Mohammad Aziz Rasouli

**Affiliations:** 1 Department of Prosthodontics, Faculty of Dentistry, Kurdistan University of Medical Sciences, Sanandaj, Iran; 2 Social Determinants of Health Research Center, Research Institute for Health Development, Kurdistan University of Medical Sciences, Sanandaj, Iran; 3 Dentistry Student, Faculty of Dentistry, Student Research Committee, Kurdistan University of Medical Sciences, Sanandaj, Iran; 4 DDS & Fellowship Oral Implantology (ICOI Fellowship), Faculty of Dentistry, Kurdistan University of Medical Sciences, Sanandaj, Iran; 5 Vice Chancellor for Educational and Research, Kowsar Hospital, Kurdistan University of Medical Sciences, Sanandaj, Iran

**Keywords:** Knowledge, Attitude, Practice, Hepatitis B, Hepatitis C, HIV/AIDS, Dentists, Iran

## Abstract

**Background:**

Healthcare workers including physicians, dentists, nurses and laboratory workers are considered to be among the groups at the risk of blood-borne pathogen transmission. Thus, it is necessary to evaluate the Knowledge, Attitude, and Practices (KAP) of dentists regarding infection control and basic principles.

**Methods:**

This cross-sectional study recruited 106 dentists in Sanandaj, Iran. The dentists’ KAP regarding hepatitis B virus (HBV), hepatitis C virus (HCV), and HIV/AIDS were evaluated. Chi-square test, student’s t-test, and one-way ANOVA were used to assess differences between the groups. Data were analyzed in Stata 12.

**Results:**

The results showed that the majority of the subjects in the study population (53.8%) were female. The mean ± standard deviation (*SD*) for age and work experience was 39.6 ± 9.80 and 10.6 ± 8.7 years, respectively. The mean ± SD for knowledge, attitude and practices of participants was 37.3 ± 3.01, 22.9 ± 4.80 and 24.07 ± 5.06, respectively. The results also indicated that dentists’ higher level of knowledge about HBV, HCV and HIV/AIDS was significantly influenced by work experience (≥10 years; *P* < 0.001) and graduation year (after 2006: *P* < 0.001). Positive attitude towards HBV, HCV and HIV/AIDS was considerably influenced by age group (< 30 years: *P* = 0.021), work experience (≥10 years: *P* < 0.001), and workplace (dental office: *P* = 0.016).

**Conclusions:**

The results of this study demonstrated a satisfactory level of knowledge and attitude of dentists about HBV, HCV and HIV/AIDS infections, but some gaps were observed, suggesting that higher knowledge level of dentists plays a very important role in forming the attitudes and practices regarding patients with HBV, HCV and HIV/AIDS.

**Electronic supplementary material:**

The online version of this article (10.1186/s12903-018-0685-1) contains supplementary material, which is available to authorized users.

## Background

According to the estimates published by the World Health Organization (WHO), 36.7 million people were infected with HIV by the end of 2015, showing an increasing trend in Iran in recent years [1]. Known to be serious health problems worldwide, the HBV and HCV infections are the commonest severe liver diseases to which all members of the society are naturally susceptible [2, 3]. Although HBV is a vaccine-preventable disease, no safe and effective HBV vaccine has yet been developed [4]. The overall prevalence of HBV is less than 1% [5, 6].

WHO estimates that two million injuries cause about 66,000 HBV, 16,000 HCV and about 1000 (200–5000) HIV infections among 35 million healthcare workers each year [7, 8]. HBV, HCV and HIV/AIDS infections remain the major global public health problems. Healthcare workers (HCWs) are at a higher risk of contracting HBV infection from infected patients. WHO reported the annual prevalence of injuries by sharp instrument and needle stick to be 4 per each HCW in Asia [9–11]. Approximately, 14.4 and 1.4% of HBV and HCV infections have been reported in HCWs, respectively, the highest prevalence being among dentists, nursing staff, dialysis unit staff, laboratory staff, and physicians [12]. Dentists are more prone to different infections. Healthcare centers such as dental clinics lack effective infection control practices [13, 14]. Several studies investigated the knowledge about principles of infection control among dentists, laboratory technicians and students. The results of these studies showed poor knowledge of dentists about principles of infection control [15–18].

However, previous studies conducted in Iran showed that dentists have a good knowledge of ways of HBV and HCV transmission, and methods of protection and prevention after exposure to HIV [19]. Given the increasing rate of blood-borne infections, especially HIV/AIDS, and the economical and psychological issues regarding job injuries, it is important to implement an effective and immediate training intervention for healthcare personnel. The training intervention should include the mode of transmission, infection mechanisms of pathogenic organisms, methods of prevention and control, and appropriate actions in response to contamination and injuries by sharp instruments [20, 21].

Naturally, limited knowledge of the community and groups at risk is the greatest obstacle to having prevention and monitoring programs. As an important measure, accurate and effective knowledge could be imparted to people using the methods that conform to the beliefs and culture of the society. Thus, the current study was conducted on the dentists’ KAP in Sanandaj regarding principles of infection control for HBV, HCV and HIV/AIDS.

## Methods

This cross-sectional study was conducted on 106 dentists in Sanandaj, Kurdistan province, western Iran. All the dentists working in private and public clinics, or dental offices in Sanandaj were recruited. The questionnaire’s validity and reliability were confirmed in other studies [22–25]. The questionnaire was designed to obtain the demographic data, work experience, graduation year and workplace profile of the dentists. ‘An additional file shows this in more detail (see Additional file 1)’. This study was approved by the Ethics Committee of the School of Dental Medicine, Kurdistan University of Medical Science.

The questionnaire consisted of four major parts: demographic information (age, sex, work experience, and educational level), and the KAP regarding HBV, HCV and HIV/AIDS infections. The number of questions related to knowledge, attitude and practice was 16, 13 and 17, respectively. Sixteen questions had three possible responses: yes, no, and do not know (each correct answer received three points, two points for “do not know” answers, and one point for incorrect answers). Thirteen items on the attitude toward these infections were assessed using a three-point Likert scale (3 = agree, 2 = uncertain, 1 = disagree), and 17 items on practice were scored with 4 responses (always, often, sometimes and never). Descriptive statistics including percentage, mean and SD of scores were used and standardized scores (score percentages of maximum possible scores) were calculated in each category. The scores were stratified into poor, medium and good categories and the correlations between the categorized scores were assessed. Good knowledge was defined as correct answers to > 12 questions, average knowledge 8–12 questions, and poor knowledge < 8 questions. The attitude was categorized into negative (scores 13–21), neutral (scores 22–30) and positive attitude (scores 31–39). The practice was also categorized into poor (scores 18–36), moderate (scores 37–54) and good practice (scores 55–72).

Chi-square test, student’s t-test, and one-way ANOVA were used to assess differences between the groups and Spearman’s rank correlation coefficient. Data were analyzed in Stata 12. The significance level in this study was considered to be *P* < 0.05.

## Results

A total of 106 dentists were selected, 53.8% of whom were female and 52.8% had graduated after 2006. Most of the subjects were employed in the public-private clinics. The mean ± SD of age and work experience were 39.6 ± 9.8 and 10.6 ± 8.7 years, respectively. The scores of knowledge, attitude, and practice regarding HVB, HCV and HIV/AIDS infections were 37.3, 22.9 and 45.2, respectively (Table 1).

**Table 1:** Comparison of knowledge, attitudes and practices regarding HBV, HCV, and HIV/AIDS infections among dentists in terms of age, sex, work experience, graduation year and workplace (*N* = 106)

Demographic characteristics	Knowledge	P*	Attitudes	P*	Practices	P*
Dentists KAP Levels		–		–		–
Negative (< 50%)	17–28		13–21		18–36	
Neutral (50–70%)	29–40		22–30		37–54	
Positive (> 70%)	41–51		31–39		55–72	
Age (year)	Mean ± SD	0.772	Mean ± SD	0.021	Mean ± SD	0.793
< 30	3.6 ± 37.3		3 ± 27.4		6.7 ± 44.5	
31–40	3.5 ± 37.1		4.1 ± 22.3		5.1 ± 45.1	
> 40	2.3 ± 37.6		4.1 ± 22.9		3.7 ± 45.6	
Sex		0.188		0.315		0.829
Male	2.7 ± 37.5		4.2 ± 22.7		4.8 ± 45.4	
Female	3.2 ± 37.2		4 ± 23.1		4.8 ± 45.1	
Work Experience		< 0.001		0.775		0.876
<10 years	3.5 ± 34.4		3.7 ± 22.3		5.6 ± 45.2	
≥ 10 years	2.1 ± 37.2		4.1 ± 23.5		3.3 ± 48.3	
Graduation Year		< 0.001		0.619		0.439
Before 2006	2.1 ± 33.4		4 ± 23.6		3.8 ± 45.6	
After 2006	3.6 ± 37.3		4.1 ± 22.5		5.9 ± 44.7	
Workplace		0.101		0.016		0.421
Public clinics	3.5 ± 38		4.2 ± 22.3		12.7 ± 42	
Private clinics	2.9 ± 37		3.7 ± 24		5.6 ± 46	
Dental offices	2.1 ± 39.2		3.7 ± 24.8		3.6 ± 45.7	
Public & private clinics	3 ± 36.4		3.7 ± 22.1		3.9 ± 46.2	

^*^Significant with ANOVA results (*P* ≤ 0.05)

The majority of the subjects showed the relatively high level of knowledge on the HBV, HCV and HIV/AIDS transmission and treatment methods. For example, their knowledge of HIV/AIDS transmission methods was as follow mother-to-child (92.6%), air- or water-borne (87.7%). Also, their knowledge on infection transmission through social behaviors such as kissing, shaking hands, and sharing glasses was 77.4%. The lowest scores of knowledge were about HBV prevention and vaccination after needle injuries, as only 11.3% provided correct answers (Table 2).

**Table 2:** Items on knowledge about HBV, HCV, and HIV/AIDS infections among dentists (*N* = 106)

Questions	Yes N (%)	Do Not Know N (%)	No N (%)	Correct Answer
Can HIV/AIDS be transmitted from mother to child?	102 (92.6)	–	4 (8.4)	102
Can HIV/AIDS be transmitted through air or water?	11 (10.3)	2 (1.9)	93 (87.7)	93
Can HIV/AIDS be transmitted through social contact (shaking hands, kissing, sharing glasses, clothes, etc.)?	20 (18.8)	4 (3.8)	82 (77.4)	82
Can HIV/AIDS be transmitted through saliva?	75 (70.8)	5 (4.7)	26 (24.5)	75
Can HIV/AIDS be completely cured with antiretroviral therapy?	85 (80.2)	4 (3.8)	17 (16)	17
Can antiviral medications (e.g. acyclovir, amantadine) be used to treat HIV/AIDS?	32 (30.2)	28 (26.4)	46 (43.4)	46
Can patients with HIV/AIDS donate blood?	13 (12.3)	13 (12.3)	80 (75.5)	80
Is post-exposure HIV/AIDS prophylaxis recommended after a needlestick injury?	89 (84)	17 (16)	–	89
Can HIV infection develop into AIDS within a year?	70 (66)	12 (11.3)	24 (22.6)	24
Is the risk of HIV/AIDS infection after a needlestick about 50–75%?	22 (20.8)	9 (8.5)	75 (70.8)	22
Is HBV mainly transmitted through sexual contact or blood?	84 (79.2)	14 (13.2)	8 (5.7)	84
Is a vaccine for HCV available?	70 (66)	11 (10.4)	25 (23.6)	70
Should individuals with HBV and HCV infections receive dental treatment in hospital?	42 (39.6)	15 (14.2)	49 (46.2)	49
Is the risk of HCV infection after a needlestick about 10–20%?	16 (15.1)	55 (51.9)	35 (33)	35
Is vaccination against HBV an efficient protection against infection after an infected needlestick?	12 (11.3)	23 (21.7)	71 (67)	12
Is transmission after needlestick higher for HBV in comparison with HIV/AIDS?	56 (52.8)	31 (29.3)	19 (17.9)	56

Also, most dentists reported positive attitudes toward HBV, HCV and HIV/AIDS. For instance, the transmission risk of HBV, HCV and HIV/AIDS from patient to patient, patient to dentist and dentist to patient without taking clinical precautions was 90.6, 88.7 and 80.2%, respectively. Also, 64.2% of the participants agreed that patients with HBV, HCV and HIV/AIDS infections should receive dental services from special clinics (Table 3).

**Table 3:** Items on attitude towards HBV, HCV and HIV/AIDS among dentists (N = 106)

Statement	Agree N (%)	Uncertain N (%)	Disagree N (%)
I would prefer not to treat patients who are HIV/AIDS positive.	26 (24.5)	29 (27.4)	50 (47.2)
Dentists should have the opportunity to refuse to treat patients with HBV, HCV and HIV/AIDS.	24 (22.6)	11 (10.4)	71 (67)
Patients with HVB, HCV and HIV/AIDS should receive dental treatment in specialized clinics.	68 (64.2)	8 (7.5)	30 (28.3)
If I found out that my longtime patient had HBV, HCV and HIV/AIDS, I would stop treating him.	15 (14.2)	15 (14.2)	76 (71.6)
Fear and concern about being infected with HVB, HCV and HIV/AIDS is one of the reasons to refuse infected patients.	31 (29.2)	32 (30.2)	43 (40.6)
Dentists are anxious about increasing the transmission risk of the HBV, HCV and HIV/AIDS while treating them.	40 (37.7)	38 (35.8)	28 (26.4)
Regardless of clinical precautions, there is risk for HIV/AIDS and hepatitis transmission from patient to dentist.	98 (88.7)	9 (8.5)	3 (2.8)
Regardless of clinical precautions, there is a risk for HIV/AIDS and hepatitis transmission from dentist to patient.	85 (80.2)	4 (3.8)	17 (16)
Regardless of clinical precautions, there is a risk for HIV/AIDS and hepatitis transmission from patient to patient.	96 (90.6)	6 (5.7)	4 (3.8)
Dentists have a professional obligation to treat HIV/AIDS positive patients.	70 (66)	26 (24.5)	10 (9.4)
Infection control measures for preventing HIV/AIDS transmission should be more than those for the prevention of HBV and HCV	44 (41.5)	17 (16)	45 (42.5)
Infection control principles are adequate for preventing the HBV, HCV and HIV/AIDS transmission.	58 (55.7)	16 (15.1)	31 (29.2)
All patients should be considered potentially infectious.	80 (75.5)	26 (24.5)	–

Table 4 presents the practice scores of dentists. About 93.4% believed in using latex gloves, and 91.5% believed in sterilization by dry heat and autoclave equipment.

**Table 4:** Items on practice regarding HBV, HCV and HIV/AIDS among dentists (N = 106)

Statement	Always N (%)	Often N (%)	Sometimes N (%)	Never N (%)
Using latex gloves	99 (93.4)	7 (6.6)	–	–
Changing gloves between patients	90 (84.9)	16 (15.1)	–	–
Using facemask	94 (84.7)	12 (11.3)	–	–
Changing face mask between patients	58 (54.7)	39 (36.8)	8 (7.5)	1 (0.9)
Using gown	58 (54.7)	21 (19.8)	16 (15.1)	11 (10.4)
Washing hands before treatment	72 (67.9)	19 (17.9)	14 (13.2)	1 (0.9)
Washing hands after treatment	87 (82.1)	13 (12.3)	5 (4.7)	1 (0.9)
Changing dental unit cover daily	64 (60.4)	27 (25.5)	9 (8.5)	6 (5.7)
Using protective glasses	85 (80.1)	15 (14.2)	6 (5.7)	–
Washing protective glasses	84 (79.2)	21 (19.8)	1 (0.9)	–
Covering all instruments to prevent contamination	79 (74.5)	25 (23.6)	2 (1.9)	–
Recapping needles	88 (83)	17 (16)	1 (0.9)	
Using gown for patient	83 (73.8)	17 (16)	5 (4.7)	1 (0.9)
Sterilizing your instruments by autoclave or dry heat	97 (91.5)	7 (6.6)	2 (1.9)	
Accepting patients with HVB, HCV and HIV/AIDS infections	41 (38.7)	33 (31.1)	28 (26.4)	4 (3.8)
Willing to work with the centers that service the patients infected with HVB, HCV and HIV/AIDS	40.9 (37.7)	28 (26.4)	17 (16)	21 (19.8)
Existence of fear and concern during treatment of the patients with HBV, HCV and HIV/AIDS	62 (58.5)	25 (23.6)	15 (14.2)	4 (3.8)

**Fig. 1:**
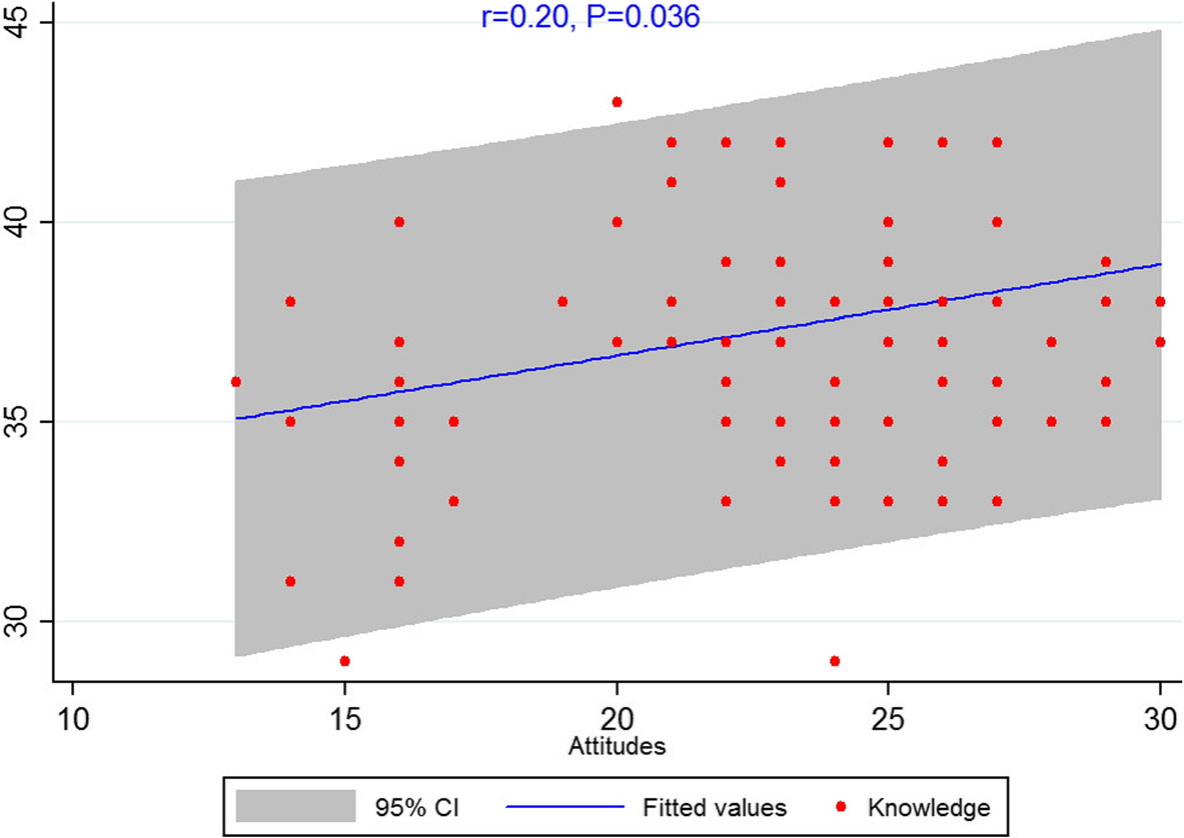
Scatterplot of scores between knowledge and attitude among dentists

**Fig. 2:**
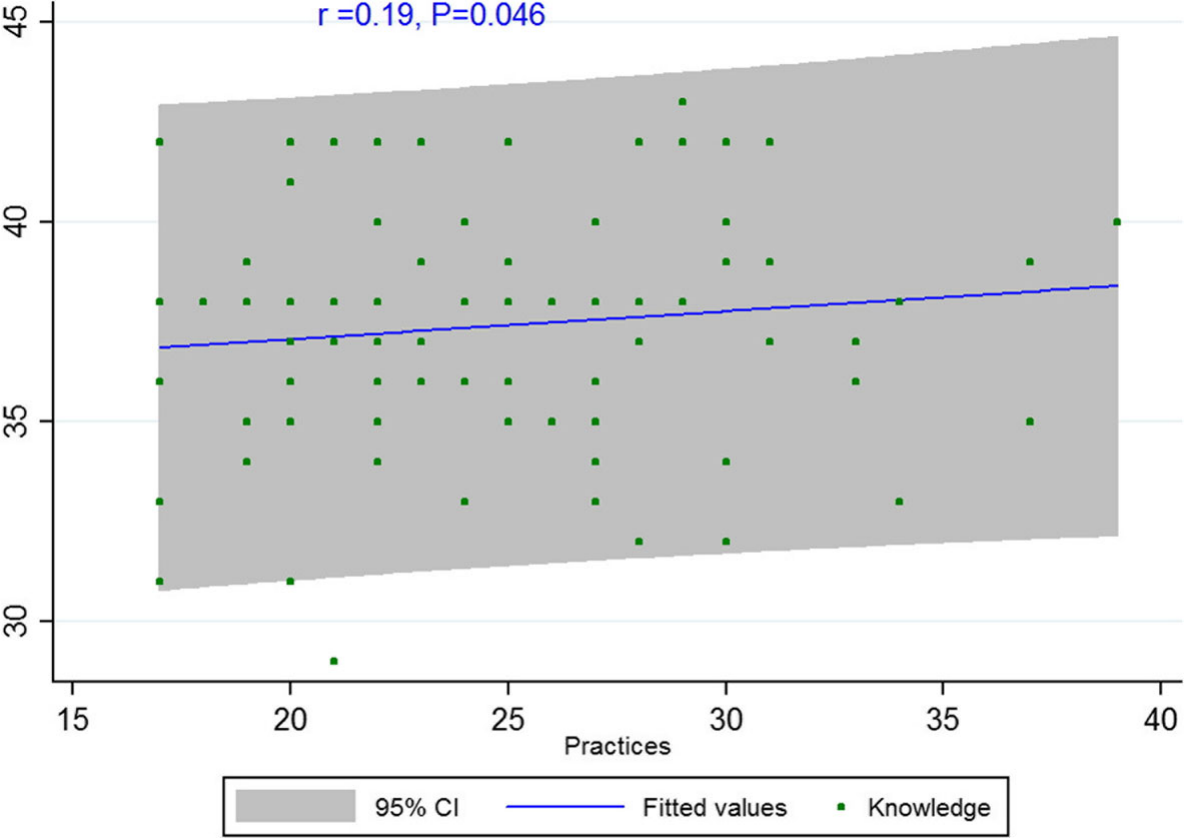
Scatterplot of scores between knowledge and practice among dentists

**Fig. 3:**
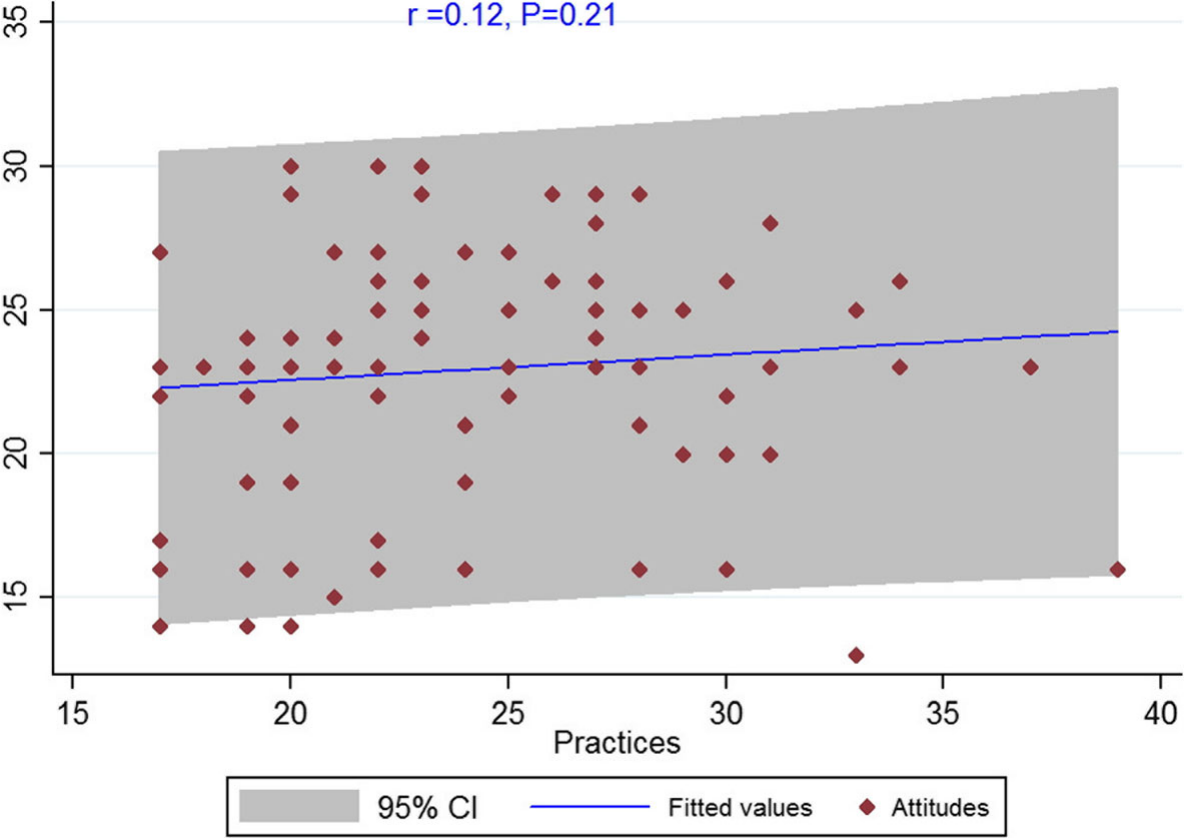
Scatterplot of scores between attitude and practice among dentists

The Spearman’s correlation showed a significant correlation between knowledge and attitude among dentists (*r* = 0.20, *P* = 0.036) (Fig. 1). There was also a significant correlation between attitude and practice (*r* = 0.19, *P* = 0.046) (Fig. 2). However, no significant correlation was observed between knowledge and practice (*r* = 0.12, *P* = 0.21) (Fig. 3).

Results showed that dentists’ higher level of knowledge about HBV, HCV, and HIV/AIDS was significantly influenced by work experience (≥10 years; *P* < .001) and graduation years (after 2006; *P* < 0.001). As for the attitude score, positive attitude towards HBV, HCV and HIV/AIDS infections was mainly influenced by age group (< 30 years: *P* = 0.021) and workplace (dental office; *P* = 0.016) (Table 1).

## Discussion

This is the first study on the KAP regarding HBV, HCV and HIV/AIDS infections in dental settings in Sanandaj. Dentists’ KAP level regarding HBV, HCV and HIV/AIDS was shown to be moderate and good. Dentists with an age less than 30 years, longer work experience, and those graduated after 2006 displayed better knowledge and attitude. Also, knowledge and attitude of dentists working in private clinics and dental settings were shown to be better compared with other dentists.

### Knowledge

Rabiee et al. [26] reported that 67 and 30% of the dentists respectively had poor and moderate knowledge about transmission and treatment methods of HIV/AIDS, HBV, and HCV infections. In case of exposure to HCV patients, the first step is to apply pressure on the wound, wash the wound area well and attempt to eliminate the infection. Also, in case of exposure to HBV, HBsAg test should be performed to ensure if the individual is a carrier or not, and immunoglobulin should be injected to injured people who are already vaccinated. Kakouei et al. [27] showed that lack of knowledge about the importance of sterilization can lead to infection transmission. Askarian et al. [23] also argued that despite the acceptance of HIV/AIDS treatment by dentists, a moderate to extremely high fear and anxiety of transmitting HIV to oneself or other patients was observed among dentists. Results from the studies on dentists revealed that the following factors were observed to play a significant role in rejecting HIV/AIDS patients by dentists: the fear of transmitting infection to their families and other personnel, the fear of losing other patients due to the fear of disease transmission from infected patients to other patients, the high expenses of recommended actions to prevent and control HIV/AIDS infection if the test is positive, and last but not least, lack of moral responsibility to treat this group of patients [28].

Leon et al. [29] showed that 93% of the subjects had no or little knowledge about the standard precautions. Hammond et al. [30] also found that only 16% of the dentists applied standard precaution guidelines. Most exposures to infected cases in dentistry are random and can be prevented through paying attention to the infection control guidelines. In some cases where contact and exposure are inevitable, timely vaccination and proper behavior can effectively prevent infection and related side effects [28]. The need for further training can be justified by the fact that dentists need a separate course that would cover theoretical and practical knowledge about patients with HBV, HCV and HIV/AIDS.

### Attitude

Based on the findings of Sadeghi [31], though, the majority of Iranian dentistry students have appropriate knowledge of HIV/AIDS. Jafari et al. investigated the knowledge and attitude of senior dental students towards HIV/AIDS infection and suggested the inclusion of training courses to promote knowledge and attitude of dental students towards HIV/AIDS in the Iranian dental curriculum [32]. Rabiee et al. [33] found that 26.3% of dentists had a negative attitude, and 73% had a positive attitude towards having contact with HIV/AIDS patients. Generally, the positive attitude of dentists towards treating high-risk patients and high level of concern for their health and risk of the virus transmission to others were consistent with the results of previous studies [26]. The higher level of knowledge may decrease individuals’ negative attitude towards HBV, HCV and HIV/AIDS infections [34].

### Practice

Based on the results of this study, we can deduce that Sanandaj dentists have a fair practice regarding HBV, HCV and HIV/AIDS infections. Ajami et al. [35] showed that 27% of the participants reported poor practice, 60% displayed moderate, and 12% revealed good practice regarding HBV, HCV and HIV/AIDS infections. Saglam et al. reported that 48.5% use gloves, whereas Burke reported 60% [36, 37]. Wearing gloves is an important protective way to avoid cross-contamination. Careless use of dental instruments can lead to increased risk of cross-contamination through rupture or hole in the glove or even hand cut. A study on infection control among dentists of Amsterdam showed that subjects paid a good attention to the use of protective coatings, mask and gloves, but less attention was paid to sterilization of the equipment [38]. In the current research, the dentists’ practice in terms of protective coatings showed more personal protective equipment usage [13, 33, 39], which may be due to the availability of this equipment. Askarian et al. [23] found that individual practice was poor towards the standard control precautions despite the acceptable knowledge and attitude of the participants, indicating that, as exposure control principles, knowledge about infection control measures and positive attitude are not enough to prevent infection; it is necessary to implement a series of continuous exposure control programs for health professionals, especially dentists, to reduce or prevent the risk of infection transmission to both dentists and patients.

The results of the present paper show no significant difference between the level of knowledge and sex, a result supported by Saber [40] and Jafari et al. [32]. Rabiee et al. [26] proposed a significant relationship in the level of knowledge between men and women: women’s knowledge level was higher than men’s.

In this study, no significant relationship was found between level of KAP and sex, while Rabiee et al. [26] showed that female dentists had a negative attitude towards the treatment of patients with HVB, HCV and HIV/AIDS. The difference could be due to the fact that greater knowledge leads to the negative attitude towards the treatment of these patients.

Results of this study showed that longer work experience was associated with higher knowledge. Rabiee et al. [33] showed a significant relationship between knowledge level and work experience. As such, higher knowledge and more work experience at older ages can result from the effect of 5-year training on the target population. Similar to previous studies, work experience was shown to have no effect on attitude and practice [39, 41].

Knowledge of dentists who graduated after 2006 was better in this study. Also, Saber et al. [40] proposed a significant relationship between the level of knowledge and graduation date among dentists, which was in compliance with our study.

Vejdani et al. [42] found no statistically significant relationship between knowledge and practice among dentists working in the public sector and those working in private clinics. Differences between the educational curriculum and culture may explain some of these differences. In Crossley’s study, the results showed that age and type of dental practice were significantly associated with treatment practice and attitude of the dentists towards HIV/AIDS infection [43]. Differences between the educational curriculum and culture may explain some of these differences.

#### Study limitations

This study does not primarily assess knowledge and attitude in the context of cross-infection risks. It also involves a limited number of samples.

## Conclusion

Moderate knowledge level of dentists’ regarding HBV, HCV and HIV/AIDS infections was influenced by work experience and year of graduation from university. Positive attitude towards infected patients was mainly influenced by workplace (private clinic and dental office). These results demonstrated a satisfactory knowledge level and positive attitude about HBV, HCV and HIV/AIDS infections amongst dentists, but some gaps were observed, suggesting that higher knowledge level of dentists plays a very important role in forming the attitudes and practices regarding patients with HBV, HCV and HIV/AIDS.

## Additional file

Additional file 1: Questionnaire. Dentists’ Knowledge, Attitudes and Practices regarding Hepatitis B and C and HIV/AIDS in Sanandaj city. (PDF 262 kb)

## Abbreviations

AIDS: Acquired immune deficiency syndrome
HBV: Hepatitis B virus
HCV: Hepatitis B virus
HCWs: Healthcare workers
HIV: Human immune-deficiency virus
KAP: Knowledge, attitudes, practices
SD: Standard deviation
STIs: Sexually transmitted infections
