# Supplementary material for: Dentists’ knowledge, attitudes and practices regarding Hepatitis B and C and HIV/AIDS in Sanandaj, Iran
Source: BMC Oral Health. 2018 Dec 18;18:220. doi: 10.1186/s12903-018-0685-1 (PMC6299669; doi:10.1186/s12903-018-0685-1)
Supplement: Supplementary file 1 — Questionnaire. Dentists’ Knowledge, Attitudes and Practices regarding Hepatitis B and C and HIV/AIDS in Sanandaj city. (PDF 262 kb) [file 12903_2018_685_MOESM1_ESM.pdf]

Questionnaire

Dentists' Knowledge, Attitudes and Practices regarding Hepatitis B and C and HIV/AIDS in Sanandaj city

Dear Dentist: This questionnaire is designed to measure the knowledge, attitude, and practice of dentists in Sanandaj city regarding hepatitis B and C and HIV / AIDS, for a student dentistry course in dentistry. Please answer the questions thoroughly. Thank you for your cooperation. Your information will remain secret to us.

……

1. Age: …….   2. Sex: a) Male   b) Female

3. Work Experience: ……Years…… Month   4. Graduation Year from University: …………

5. Workplace: a) Public clinics b) Private clinics c) Dental offices d) Public & private clinics

Items on knowledge about HBV, HCV, and HIV/AIDS infections among dentists

Questions	Yes	Do Not Know	No
1. Can HIV/AIDS be transmitted from mother to child?			
2. Can HIV/AIDS be transmitted through air or water?			
3. Can HIV/AIDS be transmitted through social contact (shaking hands, kissing, sharing glasses, clothes, etc.)?			
4. Can HIV/AIDS be transmitted through saliva?			
5. Can HIV/AIDS be completely cured with antiretroviral therapy?			
6. Can antiviral medications (e.g. acyclovir, amantadine) be used to treat HIV/AIDS?			
7. Can patients with HIV/AIDS donate blood?			
8. Is post-exposure HIV/AIDS prophylaxis recommended after a needlestick injury?			
9. Can HIV infection develop into AIDS within a year?			
10. Is the risk of HIV/AIDS infection after a needlestick about 50%-75%?			
11. Is HBV mainly transmitted through sexual contact or blood?			
12. Is a vaccine for HCV available?			
13. Should individuals with HBV and HCV infections receive dental treatment in hospital?			
14. Is the risk of HCV infection after a needlestick about 10%-20%?			
15. Is vaccination against HBV an efficient protection against infection after an infected needlestick?			
16. Is transmission after needlestick higher for HBV in comparison with HIV/AIDS?			

Items on attitude towards HBV, HCV and HIV/AIDS among dentists

Statement	Agree	Uncertain	Disagree
1. I would prefer not to treat patients who are HIV/AIDS positive.			
2. Dentists should have the opportunity to refuse to treat patients with HBV, HCV and HIV/AIDS.			
3. Patients with HVB, HCV and HIV/AIDS should receive dental treatment in specialized clinics.			
4. If I found out that my longtime patient had HBV, HCV and HIV/AIDS, I would stop treating him.			
5. Fear and concern about being infected with HVB, HCV and HIV/AIDS is one of the reasons to refuse infected patients.			
6. Dentists are anxious about increasing the transmission risk of the HBV, HCV and HIV/AIDS while treating them.			
7. Regardless of clinical precautions, there is risk for HIV/AIDS and hepatitis transmission from patient to dentist.			
8. Regardless of clinical precautions, there is a risk for HIV/AIDS and hepatitis transmission from dentist to patient.			
9. Regardless of clinical precautions, there is a risk for HIV/AIDS and hepatitis transmission from patient to patient.			
10. Dentists have a professional obligation to treat HIV/AIDS positive patients.			
11. Infection control measures for preventing HIV/AIDS transmission should be more than those for the prevention of HBV and HCV			
12. Infection control principles are adequate for preventing the HBV, HCV and HIV/AIDS transmission.			
13. All patients should be considered potentially infectious.			

Items on practice regarding HBV, HCV and HIV/AIDS among dentists

Statement	Always	Often	Sometimes	Never
1. Using latex gloves				
2. Changing gloves between patients				
3. Using facemask				
4. Changing face mask between patients				
5. Using gown				
6. Washing hands before treatment				
7. Washing hands after treatment				
8. Changing dental unit cover daily				
9. Using protective glasses				
10. Washing protective glasses				
11. Covering all instruments to prevent contamination				
12. Recapping needles				
13. Using gown for patient				
14. Sterilizing your instruments by autoclave or dry heat				
15. Accepting patients with HVB, HCV and HIV/AIDS infections				
16. Willing to work with the centers that service the patients infected with HVB, HCV and HIV/AIDS				
17. Existence of fear and concern during treatment of the patients with HBV, HCV and HIV/AIDS				
